# Data on pilot assessment of efficacy of artemether lumefantrine when co-administered with ciprofloxacin in malaria-typhoid co-infected patients

**DOI:** 10.1016/j.dib.2021.106732

**Published:** 2021-01-09

**Authors:** Segun Solomon Ogundapo, Olajoju Temidayo Soniran, Caroline Ibukun Vining-Ogu, Karian Chigozie Ngobidi, Nwogo Ajuka Obasi, Victor Uzochukwu Olugbue, Jonathan Adebanjo Adegbola, Adebimpe Foluke Ogundapo

**Affiliations:** a Department of Science Laboratory Technology, Akanu Ibiam Federal Polytechnic Unwana Afikpo Ebonyi State, Nigeria; b Department of Epidemiology Noguchi Memorial Institute for Medical Research, University of Ghana, Legon, Ghana; c Department of Medical Biochemistry Alex Ekwueme, Federal University Ndufu Alike Ikwo, Nigeria; d Department of Pharmaceutical Chemistry Obafemi, Awolowo University Ile Ife, Nigeria; e Medical Centre, Akanu Ibiam Federal Polytechnic Unwana Afikpo Ebonyi State, Nigeria

**Keywords:** Malaria-Typhoid, Day 7 Lumefantrine, Efficacy, Artemether lumefantrine, Ciprofloxacin

## Abstract

Malaria -typhoid co-infection is associated with poverty and underdevelopment with significant morbidity and mortality with similarities in clinical features of the two diseases that often result in misdiagnosis and mistreatment of the febrile patients. The Co-administration of artemether lumefantrine (AL) with ciprofloxacin as treatment for malaria-typhoid co-infection is common in Nigeria and this increases risk of pharmacokinetic drug-drug interaction since ciprofloxacin is an inhibitor of CYP3A4 that metabolizes AL. In an open-label prospective three arm design with registration pactr201909811770922, one hundred and nineteen (119) febrile volunteers comprising 55 males and 64 females were distributed into three oral treatment regimen groups. Group 1 consist of sixty-five participants presenting malaria mono infection treated with AL only and fifty-four participants presenting malaria-typhoid co-infection randomly assigned to Group 2 treated with AL and ciprofloxacin concomitantly and Group 3 whose doses were staggered at 2 hours interval. Blood samples were collected from participants in the three groups on 3 different days: day 0 (before commencement of treatment); day 3 (after completion of AL); and day 7 (after completion of ciprofloxacin), The collected blood sample were used to determine parasite density, serum liver and kidney function parameters, haematological indices, and day 7 lumefantrine concentration. The data in this article provides the changes in PCR-uncorrected Early Treatment Failure (ETA), Late Clinical Failure (LCF), Late Parasitological Failure (LPF), Day 7 serum lumefantrine concentration, liver and kidney function parameters, axillary body temperature and PCV/Hb associated with the different treatment regimen. The dataset [1] as a baseline, will stimulate the need for a randomized clinical assessment of the efficacy of AL when co-administered with ciprofloxacin in the treatment regimen of Malaria-typhoid co-infection in endemic areas. Such findings are capable of influencing national treatment policy on the management of malaria-typhoid co-infection.

## Specifications Table

**Table:** 

Subject	Pharmacology, Toxicology and Pharmaceutics (General)
Specific subject area	Drug-drug Interactions in co-administration of artemether lumefantrine with ciprofloxacin in treatment of malaria-typhoid coinfection
Type of data	Table
How data were acquired	Data was obtained from structured questionnaire, thick film Microscopy and spectrophotometric assay of blood samples using Randox clinical assay kits A HPLC-UV fitted with a RP Zorbax C_18_ column (150 mm x 4.6 mm, 5µm) and a gradient elution with acetronitrile/formic acid as mobile phase was used for quantification of day 7 lumefantrine concentration.
Data format	Raw and analysed data expressed as tables and charts
Parameters for data collection	Six months of parasitological and clinical chemistry analysis (June - November, 2019). Blood samples were collected from participants who met inclusion criteria and grouped into malaria and malaria-typhoid based on thick blood film microscopy and Widal agglutination test. Classification into three groups was based on treatment regimen.
Description of data collection	Data was obtained from a three-arm open label prospective protocol. Data was obtained from analysis of blood samples on 3 different days: day 0 (before commencement of treatment); day 3 (after completion of AL); and day 7 (after completion of ciprofloxacin).
Data source location	Akanu Ibiam Federal Polytechnic Unwana, Afikpo Ebonyi State South east Nigeria, situated on latitude 5^0^.53N and longitude 7^0^.56E.
Data accessibility	Data was deposited at Mendeley repository as Mendeley Data, v2 10.17632/bb93hkxxmc.2 Direct URL to data: 10.17632/bb93hkxxmc.2

## Value of the Data

• The data provided are of value as it serves as baseline for treatment outcomes and safety parameters of artemether lumefantrine when co-administered with ciprofloxacin in the treatment regimen of malaria-typhoid co-infection.
• Biomedical or clinical researchers interested in assessing drug-drug interactions associated with co-administration of artemether lumefantrine with ciprofloxacin or any other CYP3A4 enzyme inhibiting drug will find the dataset useful as a baseline or reference dataset.
• The dataset serves as a pilot baseline that underscores an urgent need for a randomized clinical trial to assess the efficacy of artemether lumefantrine when co-administered with ciprofloxacin in the treatment regimen of Malaria-typhoid co-infection in endemic areas.
• This dataset provides information on the prevalence of Malaria and malaria-typhoid co-infection in south east part of Nigeria which may be useful to health policy makers in the country.

## 1 Data Description

**Fig. 1:**
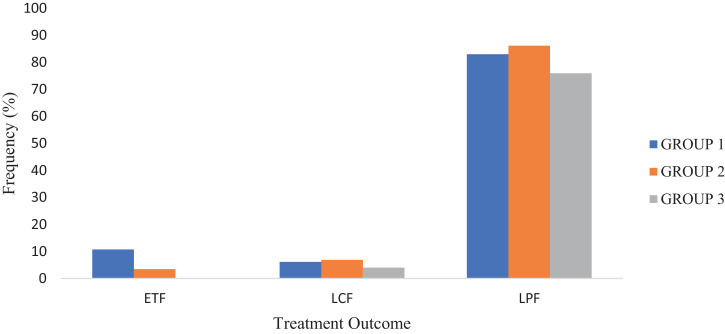
Treatment outcomes; Proportion of participants (%) with Early Treatment Failure (ETF), Late Clinical Failure (LCF) and Late Parasitological Failure (LPF) in all the groups on days 3 and 7.

**Fig. 2:**
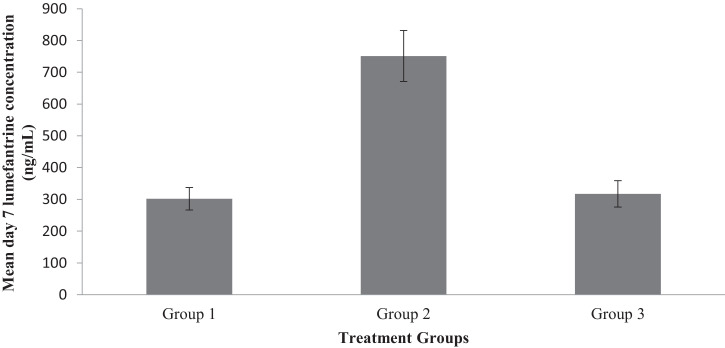
Mean day 7 lumefantrine concentration (ng/mL) in the three groups. N=15 for each group. Values are mean ± standard error of means SEM. *Mean value in Group 2 statistically significant at p < 0.05, when compared to Group 1.

This section consists of analyzed data on treatment and safety outcomes of artemether lumefantrine when it is co-administered with ciprofloxacin an inhibitor of CYP3A4 enzyme that metabolizes artemether lumefantrine to dihydroartemisinin and desbutyl lumefantrine bioactive metabolites respectively. The raw data files were deposited in Mendeley data repository [1]. Information on the demographic characteristics of the participants is given in Table 1 while that of the malaria parasite densities on days 0, 3 and 7 for all the three groups is presented in Table 2. Table 3 gives analysis of changes in parasitaemia in all the groups on days 3 and 7 while Table 4 gives information on changes in body temperature, liver and kidney function parameters and haematological indices in the participants. Fig. 1 provides information on early treatment failure (ETF), Late Clinical Failure (LCF) and Late Parasitological Failure (LPF) in all the groups on days 3 and 7 while Fig. 2 represents the changes in day 7 serum lumefantrine concentrations for the three groups. All the data files (figures, tables, raw data, dataset) are available at Mendeley data repository [1]. The structured questionnaire is provided as a supplementary file in this article while the participants’ response to the questionnaire are available as Mendeley dataset [14].

**Table 1:** Demographic characteristics of study participants*

	Malaria mono-infection (Group 1)		Malaria-Typhoid (Group 2)		Malaria-Typhoid (Group 3)	
Demographic Characteristics	Male (n=31) (%)	Female (n=34) (%)	Male (n=12) (%)	Female (n=17) (%)	Male (n=12) (%)	Female (n=13) (%)
**AGE**						
18-22	3 (9.68)	6 (17.65)	3 (25.00)	–	2 (16.67)	2 (15.38)
23-27	20 (64.56)	17 (050.00)	7 (58.53)	13 (76.47)	7 (58.33)	6 (40.15)
28-32	4 (12.90)	3 (8.82)	1 (5.88)	1 (5.88)	1 (8.33)	3 (23.07)
33-37	2 (6.45)	1 (2.94)	–	1 (5.88)	1 (8.33)	2 (15.38)
38-42	1 (3.23)	4 (11.70)	–	1 (5.88)		–
43-47	–	1 (2.94)	–	–	–	–
48-52	1 (3.23)	1 (2.94)	–	1 (5.88)		–
52 and above	–	1 (2.94)	1 (5.88)	–	1 (8.33)	–

**PROFFESSION**						
Academic staff	1 (3.23)	1 (2.94)	1 (5.88)	2 (11.76)	1 (8.33)	–
Non-academic staff	3 (9.68)	7 (20.59)	–	1 (5.88)	1 (8.33)	2 (15.38)
Students	26 (83.89)	24 (70.59)	10 (83.33)	13 (76.47).	9 (75.00)	8 (61.54)
Others	1 (3.23)	2 (5.89)	1 (5.88)	1 (5.88)	1 (8.33)	3 (23.07)

*These are the 119 study participants that were successfully followed up to day 7 consisting of 65 presenting malaria mono-infection and 54 presenting malaria-typhoid co-infection. Those presenting the co-infection were randomized into two groups based on the concomitant (29) and staggered (25) treatment regimens.

**Table 2:** Malaria parasite density of study subjects on days 0, 3 and 7 of treatment.

	GROUP 1			GROUP 2			GROUP 3		
MP Parasites/µL	n (%)	MALE n (%)	FEMALE n (%)	n (%)	MALE n (%)	FEMALE n (%)	n (%)	MALE n (%)	FEMALE n (%)
**DAY 0**									
1-999	20 (30.77)	5 (16.13)	15 (44.12)	5 (17.24)	4 (33.33)	1 (5.88)	14 (56)	8 (66.67)	6 (46.15)
1000-9999	39 (60)	23 (74.19)	16 (47.06)	19 (65.52)	8 (66.67)	11 (64.71)	10 (40)	4 (33.33)	6 (46.15)
> 10,000	6 (9.23)	3 (9.68)	3 (8.82)	5 (17.24)	0	5 (29.41)	1 (4)	0	1 (7.70)
**Total**	65	31	34	29	12	17	25	12	13

**DAY 3**									
NPD	–	–	–	–	–	–	–	–	–
1-999	44 (67.69)	23 (74.9)	21 (61.76)	17 (68.62)	8 (66.67)	9 (52.94)	19 (76)	10 (83.33)	9 (69.23)
1000-9999	20 (30.77)	8 (25.81)	12 (35.30)	12 (41.38)	4 (33.33)	8 (47.06)	6 (24)	2 (16.67)	4 (30.77)
> 10,000	1 (1.54)	0	1 (2.94)	–	–	–	–	–	–
**Total**	65	31	34	29	12	17	25	12	13

**DAY 7**									
NPD	11 (16.92)	5 (16.18)	6 (17.65)	4 (13.79)	3 (25)	1 (5.88)	6 (24)	4 (33.33)	2 (15.38)
1-999	35 (53.85)	20 (64.52	15 (44.12)	16 (55.17)	8 (66.67)	8 (47.06)	14 (56)	6 (50.01)	8 (61.54)
1000-9999	16 (24.62)	6 (19.35)	10 (29.41)	8 (27.59)	7 (8.33)	7 (41.18	4 (16)	1 (8.33)	3 (23.08)
> 10,000	3 (4.61)	0	3 (8.82)	1 (3.45)	0	1(5.88)	1 (4)	1 (8.33)	–
**Total**	65	31	34	29	12	17	25	12	13

**Group 1**= Individuals presenting malaria mono-infection treated with AL; **Group 2** = Malaria-Typhoid co-infected individuals treated with AL administered concomitantly with ciprofloxacin; Group **3**= Malaria-Typhoid co-infected individuals treated with AL and ciprofloxacin staggered at 2hours interval; **NPD**= No parasite detected.

**Table 3:** Comparative analysis of changes in parasitaemia in all the groups on days 3 and 7.

	Frequency		
Parameters	(Group 1)	(Group 2)	(Group 3)
**DAY 3 Parasitaemia**			
Cleared	–	–	–
Decreased	35/65 (53.85%)	17/29 (58.62%)	6/25 (24.00%)
Unchanged	26/65 (40.00%)	11/29 (37.93%)	19/25 (76.00%)
Increased	4/65 (6.15%)	1/29 (3.45%)	–

**Day 7 Parasiteamia**			
Cleared	11/65 (16.92%)	4/29 (13.79%)	6/25 (24.00%)
Decreased	26/65 (40.00%)	17/29 (58.62%)	5/25 (20.00%)
Unchanged	18/65 (27.69%)	7/29 (24.14%)	13/25 (52.00%)
Increased	10/65 (15.38%)	1/29 (3.45%)	1/25 (4.00%)

**Group 1**= Individuals presenting malaria mono-infection treated with AL; **Group 2** = Malaria-Typhoid co-infected individuals treated with AL administered concomitantly with ciprofloxacin; Group **3**= Malaria-Typhoid co-infected individuals treated with AL and ciprofloxacin staggered at 2hours interval

**Table 4:** Body temperature, liver and kidney function parameters and haematological in the study subjects.

	AL Treatment			AL + Cipro (Concomitant)			AL+ Cipro (Staggered)		
PARAMETERS	Group Mean	Males	Females	Group Mean	Males	Females	Group Mean	Males	Females
Body Temperature	36.50 ± 0.11	36.47 ± 6.16	36.26±0,11	36.83±0.26	37.76 ± 0.43	36.28 ± 0.31	36.18±0.16	36.07 ± 0.13	36.28 ± 6.29
	**36.53 ± 0.12**	**36.41 ± 0.16**	**36.16 ± 0.16**	**36.59 ± 0.24**	**35.94 ± 0.36**	**36.69 ± 0.23**	**36.87 ± 0.18**	**35.92 ± 0.24**	**36.75 ± 0.20**

**Liver function**									
ALT (IU/L)	32.25±2.46	33.89±3.68	30.31±3.39	17.28±1.43	16.83 ± 1.42	17.59 ± 2.28	18.20±1.45	16.35 ± 1.50	19.92 ± 2.38
	**31.42 ± 3.42**	**20.46 ± 2.06**	**41.42 ± 5.89**	**75.68 ± 12.45^b^**	80.10 ± 23.91*	**72.56 ± 13.57^a^**	**21.10 ± 2.92**	**27.83 ± 5.40**	**14.86 ±89.80**
AST (IU/L)	60.37±5.77	49.36±7.22	70.41±8.39	65.29±7.33	86.58 ± 11.79	50.26 ± 7.67	80.68±7.48	87.08 ± 111.12	74.77 ± 10.21
	**97.27 ± 6.92^a^**	**109.41 ± 9.53^a^**	**80.19 ± 9.72**	**116.42 ± 12.08^a^**	**118.58 ± 16.11^a^**	**114.89±17.58^a^**	**38.03 ± 3.11^a^**	**32.67 ± 4.07^a^**	**42.98 ± 4.38**
ALP (IU/L)	82.12±6.51	95.34±11.28	72.50±6.33	78.87±7.23	102.17 ± 10.76	59.48 ± 8.22	94.34±9.74	82.05 ± 11.74	105.69 ± 15.05
	**58.36 ± 4.57**	**54.07 ± 4.13**	**64.24 ± 7.36**	**42.22 ± 2.77^a^**	**40.78 ± 4.44**	**49.12 ± 3.35**	**135.20±11.33^a,b^**	**131.00 ± 14.60^a^**	**139.08 ± 17.62**
Total Bilirubin (mmol/l)	0.97±0.07	1.08±0.10	0.87 ± 0.09	0.99±0.10	0.84 ± 0.14	1.09 ± 0.13	0.98 ± 0.12	0.94 ± 0.09	1.01 ± 0.17
	**1.02 ± 0.08**	**0.91 ± 0.08**	**1.11 ± 0.12^a^**	**1.08 ± 0.10**	**1.12 ±++- 0.13**	**1.12 ± 0.13**	**1.02 ± 0.10**	**0.87 ± 0.17**	**1.17 ± 0.12**
Direct bilirubin (mmol/l)	0.82±0.08	0.78±0.12	0.86±0.17	0.86±0.21	0.56 ± 0.11	0.56 ± 0.11	0.56 ± 0.13	0.52 ± 0.09	0.61 ± 0.10
	**1.03 ± 0.09**	**1.25 ± 0.21**	**0.99 ± 0.13**	**1.02 ± 0.12**	**0.78 ± 0.16**	**1.18 ± 0.21**	**1.16 ± 0.25**	**1.19 ± 0.21**	**0.72 ± 0.19**

**Kidney function**									
Creatinine (µmol/l)	64.81±2.60	67.22±3.58	62.62±3.74	64.26±3.71	70.76 ± 6.38	59.68 ± 4.26	62.27 ± 4.01	60.98 ± 5.23	63.46 ± 6.22
	**58.40 ± 2.27**	**59.43 ± 3.45**	**57.46 ± 3.03**	**66.25 ± 3.73**	**70.49 ± 6.32**	**63.27 ± 4.55**	**57.65 ± 2.94**	**59.02 ± 4.79**	**56.41 ± 3.67**
Urea (mg/dl)	11.27±0.09	11.30±0.57	11.32±0.13	11.50±0.13	11.46 ± 0.19	11.53 ± 0.18	11.56 ± 0.13	11.64 ± 0.23	11.49 ± 0.15
	**11.47 ± 0.01**	**11.36 ± 0.13**	**11.56 ± 0.15**	**11.68 ± 0.73**	**11.88 ± 0.20**	**11.55 ± 0.24**	**11.18 ± 0.15**	**11.19 ± 0.24**	**10.17 ± 0.21^a^**

**Haematological indices**									
PCV	38.28±0.69	41.06 ± 8.91	35.74 ± 18.39	37.03±0.75	35.75 ± 1.38	37.94 ± 7.79	41.04 ± 0.91	43.17 ± 1.45	39.08 ± 8.36
	**39.35 ± 0.58**	**40.94 ± 0.76**	**37.91 ± 0.80**	**36.17 ± 0.94**	**35.75 ± 1.83**	**36.47 ± 0.98**	**39.48 ± 1.37**	**42.08 ± 2.25**	**37.08 ± 1.37**
Haemoglobin (g/dL)	13.82 ± 0.19	13.47±0.16	13.26 ± 0.11	13.48±0.21	13.12 ± 0.43	13.73 ± 0.31	14.23 ± 0.30	13.07 ± 0.28	13.28 ± 0.28
	**14.04 ± 0.17**	**14.08 ± 0.23**	**13.63 ± 0.21**	**13.43 ± 0.24**	**13.43 ± 0.25**	**13.43**± **0.25**	**14.12 ± 0.39**	**14.80 ± 0.66**	**13. 49 ± 0.38**

Values are Mean ± SEM. Values in **bold** represent post treatment values. Values with superscript ‘a’ are significant at p < 0.05 when compared with pre-treatment values while those with ‘b’ superscript are significant at p < 0.05 when compared with control Group 1.

## 2 Experimental Design, Materials and Methods

The protocol for data collection is based an open-label prospective three arm design with registration pactr201909811770922. The main criterion for the classification of the participants into the two groups of Malaria and malaria-typhoid co-infection is clinical laboratory diagnosis based on Giemsa thick blood film microscopy and Widal antigen agglutination test. The malarial typhoid co-infected participants where further divided into two treatment sub-groups; those treated with AL orally co-administered with ciprofloxacin concomitantly (Group 2) and Group 3 treated with AL orally co-administered with ciprofloxacin at 2 hours interval. The doses were staggered by 2 hours in order assess the effect of staggering the dosage of the two drugs on the efficacy of AL on the hypothetical drug-drug interaction. This is based on the assumption that maximum gastrointestinal absorption and peak plasma concentration of artemether would have been achieved before the administration of ciprofloxacin since the short acting component of AL is absorbed within that time frame.

## 3 Area/location

The dataset was collected between June and November, 2019 at the Akanu Ibiam Federal Polytechnic Unwana Medical Centre located in Afikpo North Local Government Area of Ebonyi state, Nigeria. The Centre serves the general medical needs of the entire polytechnic staff, students, staff dependents and patients living in and around the polytechnic community environment. Afikpo, situated on latitude 5^0^.53N and longitude 7^0^.56E is the second largest city in Ebonyi State. The average temperature in the area is highest in the month of March (84.00F, 29.0° C) and lowest in the month of August with 78.10F (25.6°c). Annual rainfall ranges from 1600m to over 2000m and the driest month having less than 29m of rainfall. The population is estimated at 156,611 [2].

## 4 Recruitment Criteria of Participants

Inclusion criteria were adults of both sexes between 18 and 60 years of age presenting symptoms like fever, head ache, vomiting, and weakness of the body, body pains and other symptoms indicating uncomplicated malaria and/or typhoid infection. Individuals who had taken antimalarial drug and/or antibiotics or herbal concoction within 2 weeks prior to the time of recruitment, those under treatment for any underlying diseases like diabetes, sickle cell anaemia, hepatitis, high blood pressure and those whose medical history suggest reduced susceptibility or sensitivity to ciprofloxacin were excluded. The goal was explained to the participants and they gave written consent before participating. The researchers took measures to ensure confidentiality to all individuals who participated. All collected samples and the filled confidential report forms were assigned codes as identification number.

## 5 Sample Collection

Blood samples were collected from participants on 3 different days: day 0 (before commencement of treatment); day 3 (after completion of AL); and day 7 (after completion of ciprofloxacin), to determine changes in parasite density, serum liver function, kidney function, haematological indices, and lumefantrine concentration. On days 0 and 7, 5mls of blood were collected aseptically by vein puncture from each participant and labeled aseptically while on day 3, drops of peripheral blood were collected by finger prick method.

## 6 Drugs and Administration

The drugs administered for the treatment of malaria mono-infection and malaria-typhoid co-infection is the generic artemether lumefantrine and ciprofloxacin dispensed at the Akanu Ibiam Federal polytechnic Medical Centre. The AL is fixed dose combination 80mg/480mg artemether lumefantrine. For the participants presenting uncomplicated falciparum malaria mono-infection Group 1, the first two doses of AL were administered at an interval of 8 hours while the subsequent four doses were taken at 12-hour interval for 3 days. For the volunteers presenting malaria-typhoid co-infection, the generic 500mg ciprofloxacin was orally co-administered with AL in Group 2 while in Group 3, ciprofloxacin was administered 2 hours after AL until the third day when the six dose 3 days artemether lumefantrine regimen was completed. Thereafter, the oral administration of the ciprofloxacin continued till day 7.

## 7 Determination of Malaria Parasite Density

Malaria parasite density was determined from Giemsa-stained thick blood films prepared using peripheral blood collected from finger pricking. The prepared thick films were examined under the microscope using × 100 oil immersion objective lens. Level of parasitaemia was calculated in microliter (μl) of thick blood film preparation and graded as: low + (1 to 999/μl), moderate ++ (1000 to 9999/μl) and high +++ (> 10,000/μL) according to WHO [3].

## 8 Examination of Blood sample using Widal Test

The Widal agglutination test was performed on all blood samples by the rapid slide titration method using commercial antigen suspension for somatic (O) and flagella (H) antigens. Exactly 50μl of the blood serum was placed on eight rows of circles on the test tiles and a drop of positive and negative serum suspension were placed beside each sample on the test slide. Antibody titer of ≥1: 80 against O and H antigen of *Salmonella typhi* was taken as a cut of value [4,5].

## 9 Determination of Biochemical and Haematological Parameters

Randox Assay kits were used for determination of the biochemical markers of tissue toxicity and used according to manufacturer's instructions. The kits include Alanine amino Transferase (ALT) with Cat. No AL100, Aspartate Amino Transferase (AST) with Cat. No AS101), Alkaline phosphatase (ALP) with Cat. No AP 542, Bilirubin with Cat. No BR 411, Creatine with Cat. No CR 510 and Urea with Cat. No UR 1068. Serum ALT, AST, ALP, Bilirubin, creatine and urea kits were based on the methods by Reitman and Frankel [6], Engelhardt et al. [7], Sherlock [8], Bartels and Bohmer [9] and urease-Berthelot method of Wheatherburn [10] respectively. Packed Cell Volume (PCV) was determined as described by Ochei and Kolhakter, [11] and haemoglobin by the relationship derived by Turkson and Ganyo [12].

## 10 Determination of Blood Lumefantrine Concentration

A HPLC-UV fitted with a RP Zorbax C_18_ column (150 mm x 4.6 mm, 5µm) and a gradient elution with acetronitrile/formic acid as mobile phase was used for quantification of lumefantrine. Samples were pretreated using a step protein precipitation by adding 50 µL of pyrimethamine (200 µg/mL) as an internal standard (IS) to a 250 µL aliquot of the plasma samples, vortex-mixed briefly, thereafter, 700 µL of cold acetonitrile was added to precipitate plasma protein. The mixture was vortex mixed for 1 min and centrifuged for 10 mins and 20 µL of clear supernatant from each sample was injected for analysis. The calibration curves fit linear regressions over the range of 50–20 000 ng/mL for lumefantrine based on the peak area ratios generated from analyte/IS peak response. The lower limit of quantification (LLOQ) for lumefantrine was 50 ng/mL. The average inter-day accuracy and precision of the lumefantrine assay in plasma were observed to range between 85.37%and 105.9%, and 4.58% and 7.21%, respectively. The average inter-day accuracy and precision of the lumefantrine assay in plasma were observed to range between 94.04% and 101.72%, and 9.38% and 11.27%, respectively.

## 11 Determination of Treatment Outcomes

The primary treatment outcome is Late Parasitological Failure (LPF) defined as presence of parasitaemia between day 7 and 28 [13] but was only assessed on day 7. Secondary treatment outcomes were classified as Early Treatment Failure (ETF) defined as presence of danger signs of severe malaria and the presence parasitaemia by day 3 greater than 25% count on day 0 according to WHO [13]. Late Clinical Failure (LCF) was determined as danger signs or severe malaria in the presence of parasitaemia between day 4 and 7 [13]. Other secondary treatment outcomes are mean day 7 lumefantrine blood concentrations, haematological indices, liver and kidney function parameters. The parasitaemia was not PCR corrected.

## 12 Statistical Analysis

Data were processed and analyzed using Statistical Package for the Social Sciences (SPSS version 17.0) software. Descriptive statistics such as frequency and percentages were used to summarize participants’ demographic characteristics and treatment outcomes. Categorical data was analyzed using Chi squared statistics. Quantitative data on changes in blood chemistry, body temperature before treatment and on day 7 withing groups was done using paired t-test. The significance of disparity of means of the blood chemistry tests between the three groups and the day 7 serum lumefantrine concentration were analyzed using one-way analysis of variance (ANOVA). P < 0.05 was considered significant.

## Ethics Statement

Institutional ethical clearance AIFPU/REG/OR/102/VOL.4/416 was obtained after a review of the research protocol by the AIFPU Medical Centre board and the polytechnic Research and Ethics Committee. Written informed consent was also sought and obtained from each of the volunteer study subjects before inclusion in the study.

## CRediT Author Statement

**Segun Solomon Ogundapo:** Conceptualization. **Segun Solomon Ogundapo, Soniran Olajoju Temidayo, Karian Chigozie Ngobidi, Ibukun Caroline Vining-Ogu, Ajuka Nwogo Obasi, Victor Uzochukwu Olugbue, Adebanjo Jonathan Adegbola, Foluke Adebimpe Ogundapo:** Methodology. **Segun Solomon Ogundapo, Foluke Adebimpe Ogundapo:** Data curation, Writing - Original draft preparation. **Soniran Olajoju Temidayo, Ajuka Nwogo Obasi, Adebanjo Jonathan Adegbola, Victor Uzochukwu Olugbue:** Reviewing and Editing.

## Declaration of Competing Interest

The authors declare that they have no known competing financial interests or personal relationships which have, or could be perceived to have, influenced the work reported in this article.

## Appendix Supplementary materials

Image, application 1
