# Supplementary material for: Data on pilot assessment of efficacy of artemether lumefantrine when co-administered with ciprofloxacin in malaria-typhoid co-infected patients
Source: Data Brief. 2021 Jan 9;34:106732. doi: 10.1016/j.dib.2021.106732 (PMC7810622; doi:10.1016/j.dib.2021.106732)
Supplement: Supplementary file 1 [file mmc1.docx]

Structured questionnaire for male and female patients attending the **Medical Centre** of **Akanu Ibiam Federal Polytechnic Unwana** (AIFPU) Afikpo and who we are inviting to participate in a research on malaria typhoid co-infection. The title of our research is **Effect of Staggered Dosing of Artemether Lumefantrin and Ciprofloxacin on Treatment Outcomes in Malaria Typhoid Co-Infection**.

FORM NO :_______________ GSM NO_________________

**SECTION A** **(FOR MALARIA INFECTION ONLY)**

Personal Information

1. **SEX**

Male Female

1. **AGE**

18-22 23-27 28-32 33-37 38-42 43-47

48-52 53 and above

1. **PROFESSION**

Academic Staff Non-academic Staff

Students None of the above

**SECTION B**

1. Have you been treated for malaria before? Yes No
2. How frequently do you treat malaria? Every month

Every 2 weeks Few times in a year I can’t remember

This is my first time

1. How do you always know that you are infected with malaria?

From laboratory test from my signs and symptoms from chemist shop

By a doctor (medical personnel) without laboratory test From friends/relatives

1. Who usually prescribe the drugs you take? Doctor Chemist shop

Self-medication Pharmacist shop

Others please specify_______________

8. Have you been treated for malaria and typhoid before? Yes No

9. Which drug do you normally take for malaria treatment? Lumatem Coartem

Amalar/Maloxine P-Alaxin Chloroquine Natural Herbs

Mixed drugs from Chemist

10. Where do you normally get or buy the anti-malaria? Chemist shop

Medical Centre Pharmacy Don’t remember

11. When was the last time you took drugs for malaria treatment? Last week Last month

Two weeks ago Last Year Others Specify please -------------

12. Which drugs do you take at the same time with anti-malarial drugs? Paracetamol

Panadol Panadol extra Multivitamin Vitamin C

Ciprofloxacin Ampiclox Amoxyl Others

Please specify----------------------

13. Do you normally finish your anti malaria drugs in the number of days the doctor, pharmacist or chemist person prescribed for you? Yes No

I can’t remember

14. If you don’t normally finish the dosage, which of the following is the reason why?

I don’t like the smell of the drugs

Once I am better I don’t need the drugs again I don’t just feel like taking drugs

15. What do you usually use to take the malaria drugs?

Water soft drinks tea juice beer/wine

Others specify please--------------------

**FOR OFFICE USE ONLY**

**Treatment regimen**

Drug(s) Administered--------------------------------------------------------

Date of start of treatment------------------------------------------------------

Date of completion of treatment----------------------------------------------

Structured questionnaire for male and female patients attending the **Medical Centre** of **Akanu Ibiam Federal Polytechnic Unwana** (AIFPU) Afikpo and who we are inviting to participate in a research on malaria typhoid co-infection. The title of our research is **Effect of Staggered Dosing of Artemether Lumefantrin and Ciprofloxacin on Treatment Outcomes in Malaria Typhoid Co-Infection**.

FORM NO :________________ GSM NO_________________

**SECTION A** **(FOR MALARIA AND TYPHOID CO-INFECTION)**

Personal Information

1. **SEX**

Male Female

1. **AGE**

18-22 23-27 28-32 33-37 38-42 43-47

48-52 53 and above

1. **PROFESSION**

Academic Staff Non-academic Staff

Students None of the above

**SECTION B**

1. Have you been treated for malaria/typhoid before? Yes No
2. How frequently have you been treating malaria/typhoid? Every month

Every 2 weeks Few times in a year Can’t remember

This is my first time

1. How do you always know that you have malaria/typhoid?

From blood test From signs and symptoms From chemist shop

By a doctor (medical personnel) without laboratory test

1. Who usually prescribe the drugs you take? Doctor Chemist shop

Self-medication Others please specify_______________

1. Which drug do you normally take for malaria-typhoid treatment? Lumatem/Ciprofloxacin Coartem/Chloramphenicol
2. lumatem/ amoxyl

Amalar/Maloxine & ciprofloxacin P-Alaxin/ ciprofloxacin lumatem/augumentin Natural Herbs

Mixed drugs from Chemist

1. Where do you normally buy the anti-malaria? Chemist shop

Medical Centre Pharmacy I Don’t remember

1. When was the last time you took drugs for malaria and typhoid treatment?

Last week Last month

Two weeks ago Last Year Others Specify please -------------

1. Which drugs do you take at the same time with anti-malarial drugs? Paracetamol

Panadol Panadol extra Multivitamin Vitamin C

Ciprofloxacin Ampiclox Others

Please specify……………………

1. Do you normally finish your anti malaria drugs in the number of days the doctor, pharmacist or chemist person prescribed? Yes No

I can’t remember

1. If you don’t normally finish the dosage, which of the following is the reason why?

I don’t like the smell of the drugs

Once I am better I don’t need the drugs again I don’t just feel like

***15.*** do you always take antimalaria with ciprofloxacine at the same time? Yes No

***16***. What do you usually use to take these drugs?

Water soft drinks tea juice beer/wine

Others specify please_____________________

**FOR OFFICE USE ONLY**

Date of start of treatment_________________________________________________

Date of completion of treatment___________________________________________

**TRETMENT REGIMEN (Please tick appropriately)**

**MTC=** Concomitant administration of artemether lumefantrin **+** ciprofloxacin

**MTS/A>C2 =** Staggered administration of artemether lumefantrin **+** ciprofloxacin (2hours)

Date expected back for laboratory check up-------------------------------
